# Identifying patterns of clinical conditions among high-cost older adult health care users using claims data: a latent class approach

**DOI:** 10.1186/s12939-022-01688-3

**Published:** 2022-06-20

**Authors:** Xiaolin He, Danjin Li, Wenyi Wang, Hong Liang, Yan Liang

**Affiliations:** 1 Department of Social Policy, Shanghai Administration Institute, Shanghai, China; 2 School of Nursing, Fudan University, Shanghai, China; 3 School of Social Development and Public Policy, Fudan University, Shanghai, China

**Keywords:** Health care costs, Older adults, Segmentation, High-cost users, Health service use

## Abstract

**Objectives:**

To identify patterns of clinical conditions among high-cost older adults health care users and explore the associations between characteristics of high-cost older adults and patterns of clinical conditions.

**Methods:**

We analyzed data from the Shanghai Basic Social Medical Insurance Database, China. A total of 2927 older adults aged 60 years and over were included as the analysis sample. We used latent class analysis to identify patterns of clinical conditions among high-cost older adults health care users. Multinomial logistic regression models were also used to determine the associations between demographic characteristics, insurance types, and patterns of clinical conditions.

**Results:**

Five clinically distinctive subgroups of high-cost older adults emerged. Classes included “cerebrovascular diseases” (10.6% of high-cost older adults), “malignant tumor” (9.1%), “arthrosis” (8.8%), “ischemic heart disease” (7.4%), and “other sporadic diseases” (64.1%). Age, sex, and type of medical insurance were predictors of high-cost older adult subgroups.

**Conclusions:**

Profiling patterns of clinical conditions among high-cost older adults is potentially useful as a first step to inform the development of tailored management and intervention strategies.

**Supplementary Information:**

The online version contains supplementary material available at 10.1186/s12939-022-01688-3.

## Introduction

Health systems worldwide are facing the challenge of increased health care costs and sustainability [1, 2]. Previous studies [3, 4] have shown that a small proportion of individuals spend the majority of health care resources. This population is referred to as high-cost health care users (also high-cost beneficiary, high user, high spender, super-utilizer) [5]. Studies have found that high-cost health care users are a heterogeneous population, and heterogeneity may contribute to the fact that few interventions have demonstrated evidence of reliable success [2, 6]. Most of these high-cost health care users are older adults [5]. Given aging populations globally and growing health care expenditures, it is important to understand the profiles of high-cost older adults.

In the literature, the definition of high-cost health care users is not consistent [5]. The commonly used operationalized criteria include [5]: high-cost health care service or technology use, such as overall health care use exceeding a fiscal threshold (e.g., top 1%/5%/10%) [6]; frequent health care service use, such as cumulative hospital length of stay, frequent emergency department visits, and multiple hospital admissions [7]; and high or complex needs, such as comorbidity profiles [8]. This may lead to the complexity in population segments of high-cost health care users.

Previous studies have supported the segmentation of high-cost health care users on the basis of prior healthcare utilization patterns [7], complex medical conditions [9], high cost [10], or a combination of these factors [11]. A concept analysis identified three main subgroups of high-cost health care users: adults with multiple chronic conditions and functional disability, the frail elderly, and patients under 65 years old with behavioral health condition or disabled [12]. Another scoping review and gap analysis focused on transitional care models for high-need, high-cost adults to reduce low-value utilization [11]. Figueroa et al. [13] identified two high-need, high-cost patient personas across 11 developed countries, using accessible patient-level datasets. In general, this heterogeneous population of high-cost health care users have received much attention recent years. To date, there has been less work published on older adults.

Recent research focused on high-cost older adults has contributed to this field using data from high or complex needs [8] or by focusing on specific populations (such as older populations with type 2 diabetes mellitus [14]) or settings (such as emergency department visits [15]). Such studies have been limited to either specified older populations, or specified settings, however, the overall characteristics of high-cost older adults are still not widely understood. Another recognized limitation of current research involves population identification [16]. Although the terminologies of “high-need” and “high-cost” are often used alternatively [11, 13, 17], high-need patients are often emphasized to target care [16], as they represent a diverse group with high burden of medical comorbidities, social complexity, functional limitations, and disability [18]. The definition of high-cost patients using health care-related expenditures may inform policy makers to optimize health policies to combat increasing medical spending. However, there is still a lack of empirically derived evidence describing the overall profiles of high-cost older adults using alternative definitions, such as exceeding a fiscal threshold.

In addition, previous work in descriptions of high-cost patients is frequently limited by methodology, such as using decision trees to assign patients to hypothesized groups, which can fail to recognize subgroups [6]. Segmentation methods, such as latent class analysis (LCA), provide an opportunity to define subgroups within a heterogeneous population [6]. LCA is widely used to cluster patients based on clinical conditions [19, 20].

With population aging and increased healthcare expenditures across the world, there is a need to further understand the overall clinical heterogeneity among high-cost older adults health care users (defined by exceeding a fiscal threshold). Identifying clinically distinct subgroups of high-cost older adults will benefit further policy design and intervention development. The purpose of this study was to define empirically derived patterns of clinical conditions among high-cost older adults using social medical insurance claims data and explore the associations between the characteristics of high-cost older adults (age group, sex, type of social medical insurance) and the patterns of clinical conditions.

## Methods

### Study population and data sources

This is a 1-year cross-sectional study designed to explore patterns of clinical conditions among high-cost older adults health care users. We analyzed data from the Shanghai Basic Social Medical Insurance Database, a large public medical insurance claims database that contains 18.4 million enrollees in Shanghai, China, including both Urban Employees’ Medical Insurance (UEMI) claims and Urban and Rural Residents’ Medical Insurance (URRMI) claims. The Shanghai Basic Social Medical Insurance had a coverage rate of more than 95% for the registered population in Shanghai in 2019. First, we defined the high-cost population as the top 1% [6] of continuously enrolled individuals according to total health care expenditures during 2019 and selected them as a cohort (*N* = 182,513).

Then, we randomly selected 2% of individuals (*N* = 3,650) in the top 1% expenditures cohort as the sample size. We used Dziak’s formula as a reference to determine sample size for latent class analysis [21]. According to the formula, when the number of items was 15, a medium effect size (w = 0.30), and a power of 0.80, the recommended sample size was 950 [21]. Considering that we might have a greater number of items than 15 in our final analysis, as well as we focused on the older adults, which approximately accounted for 40% of the registered population in Shanghai, we further expanded our target sample size to approximate 2% of the overall high-cost population (*N* = 3,650).

Finally, we included adults aged 60 years and over as the analysis sample (*N* = 2927) (see Supplementary Fig. 1). We used deidentified data including three major categories of individual-level variables: demographic characteristics, insurance types, and clinical conditions during 2019.

### Data definitions

Details in the Shanghai Basic Social Medical Insurance database include patient demographics, insurance type, date, health provider code, item of fees, primary diagnosis code, and cost. Using the original diagnostic codes extracted from the database, we created a more limited analytic data set by combining similar variables, selecting variables with high frequency and known to be important for high-cost health care utilization in China in the literature, and excluding variables that were rare or not informative. Decisions about variables were made by research team consensus (all authors). We used the International Classification of Diseases, 10th Revision (ICD-10) grouping methodology to develop indicator variables that captured the presence or absence of clinical conditions based on diagnostic codes in claims data [6]. A final list of 20 clinical conditions was generated for our latent class analysis (Supplementary Table 1). For each clinical condition variable, a patient was coded as “yes” if the corresponding diagnostic codes were present during 2019. Patients were actually assigned to the 20 clinical conditions mutually exclusively based on their primary diagnosis on a 1-year cross-sectional basis. In addition, demographic characteristics included sex and age. Insurance types were categorized as UEMI and URRMI.

### Analysis

Descriptive statistics were used to summarize participant characteristics. We used LCA to identify patterns of clinical conditions among high-cost older adults health care users. A series of LCA models ranging from two to six classes were performed for the 2927 high-cost older adults to determine latent classes in patterns of clinical conditions. The optimal model that combines goodness of fit and parsimony was selected based on various statistical fit indices and interpretability [22]. Statistical indices reported here include [23]: the Akaike Information Criterion (AIC); Bayesian Information Criterion (BIC); sample-size adjusted BIC (aBIC); Lo–Mendell–Rubin likelihood ratio test (LMR); bootstrap likelihood ratio test (BLRT); and an entropy measure. Multinomial logistic regression models were also used to determine the associations between demographic characteristics (age and sex), insurance types, and patterns of clinical conditions. LCA models were conducted in Mplus version 8.0 and all subsequent analyses were performed using Stata SE version 15.1 (StataCorp LLC, College Station, TX, USA).

## Results

### Participant characteristics

Table 1 presents the participants’ characteristics. Among the 2927 older adults, 16.6% (*N* = 486) were aged 85 years or over, with 57.9% (*N* = 1,694) aged between 75 and 84 years; 51.0% (*N* = 1,493) were women; 85.8% (*N* = 2,511) were UEMI beneficiaries.

**Table 1:** Participant characteristics (*N* = 2927)

Variable	*n* (%)
**Age (years)**	
60–74	747 (25.5)
75–84	1,694 (57.9)
≥ 85	486 (16.6)
**Sex**	
Male	1,434 (49.0)
Female	1,493 (51.0)
**Medical insurance type**	
Urban Employees’ Medical Insurance	2,511 (85.8)
Urban and Rural Residents’ Medical Insurance	416 (14.2)

### Identification of patterns of clinical conditions

Table 2 presents the results of LCA. Model fit estimates identified a six-class solution based on the lowest sample-sized aBIC (41,817.584). However, the aBIC value for a five-class solution was only slightly higher (41,928.022) compared with the six-class model, and a five-class model showed the highest entropy, representing the highest certainty of classification. Moreover, class interpretability suggested a better fit than a five-class solution. Thus, we chose a five-class model as the final solution (Table 2).

**Table 2:** LCA model fit statistics

Classes	AIC	BIC	aBIC	Entropy	LMR	BLRT
2	43,913.071	44,197.974	44,067.684	1	< 0.0001	< 0.0001
3	43,079.382	43,510.210	43,313.187	1	< 0.0001	< 0.0001
4	42,231.971	42,808.725	42,544.968	1	< 0.0001	< 0.0001
5	41,535.833	42,258.512	41,928.022	1	< 0.0001	< 0.0001
6	41,346.203	42,214.808	41,817.584	0.918	< 0.0001	< 0.0001

*AIC* Akaike information criterion, *BIC* Bayesian information criterion, *aBIC* sample-size-adjusted BIC, *LMR* *p*-value for the Lo–Mendell–Rubin likelihood ratio test, *BLRT* *p*-value for the bootstrap likelihood ratio test, *LCA* latent class analysis

The final latent classes were as follows: 1) older adults who presented malignant tumor (9.1% of participants, “malignant tumor”); 2) older adults who presented cerebrovascular diseases (10.6% of participants, “cerebrovascular diseases”); 3) older adults who presented sporadic diseases, such as lung and bronchial diseases, other types of heart disease, hypertension, diabetes, and other diseases (64.1% of participants, “other sporadic diseases”, defined as cardio-vascular and pulmonary and others in this study); 4) older adults who presented ischemic heart disease (7.4% of participants, “ischemic heart disease”); and 5) older adults who presented arthrosis (8.8% of participants, “arthrosis”). Table 3 presents the proportion of the 2927 high-cost older adults within each latent class assignment having each clinical condition category.

**Table 3:** Proportion of 2927 high-cost older adults within each latent class assignment having each clinical condition category

	Class 1	Class 2	Class 3	Class 4	Class 5
	Malignant tumor	Cerebrovascular diseases	Other sporadic diseases	Ischemic heart disease	Arthrosis
Thyroid Disease	0	0	1	0	0
Diabetes	0	0	6	0	0
Hypertension	0	0	7	0	0
Ischemic heart disease	0	0	0	100	0
Other types of heart disease	0	0	8	0	0
Cerebrovascular diseases	0	100	0	0	0
Other vascular diseases	0	0	4	0	0
Lung and bronchial diseases	0	0	11	0	0
Arthrosis	0	0	0	0	100
Spondylosis	0	0	2	0	0
Other back diseases (related to intervertebral discs)	0	0	3	0	0
Nephritis	0	0	2	0	0
Renal failure	0	0	3	0	0
Stones	0	0	1	0	0
Gastric diseases	0	0	2	0	0
Intestinal diseases	0	0	3	0	0
Liver diseases	0	0	1	0	0
Biliary and pancreatic diseases	0	0	5	0	0
Malignant tumor	100	0	0	0	0
Benign tumor	0	0	2	0	0
**Percentage of cohort**	**9.1**	**10.6**	**64.1**	**7.4**	**8.8**

### Associations between participant characteristics and patterns of clinical conditions

Table 4 presents the results of multinomial logistic regression analyses.

**Table 4:** Associations between participant characteristics and patterns of acute and chronic conditions

Characteristics	Patterns of acute and chronic conditions (other sporadic diseases)			
	Malignant tumor	Cerebrovascular diseases	Ischemic heart disease	Arthrosis
	OR (95% CI)	OR (95% CI)	OR (95% CI)	OR (95% CI)
**Age (reference: 60–74), years**				
75–84	1.89*** (1.35–2.63)	0.70* (0.53–0.94)	0.86 (0.61–1.20)	1.12 (0.81–1.53)
≥ 85	0.44* (0.23–0.83)	1.48* (1.05–2.07)	1.53* (1.02–2.31)	1.13 (0.75–1.71)
**Sex (reference: male)**				
Female	0.95 (0.73–1.23)	0.82 (0.64–1.05)	0.72* (0.54–0.97)	2.22*** (1.67–2.95)
**Medical insurance (reference: UEMI)**				
URRMI	0.60* (0.39–0.92)	0.62* (0.42–0.92)	0.52* (0.31–0.86)	1.17 (0.83–1.64)
LR chi^2^	152.09***			

*UEMI* Urban Employees’ Medical Insurance, *URRMI* Urban and Rural Residents’ Medical Insurance, *OR* odds ratio, *CI* confidence interval, *LR* likelihood ratio

^*^p < 0.05

^**^p < 0.01

^***^p < 0.001

#### Malignant tumor versus other sporadic diseases

Older adults aged 85 years or over (odds ratio [OR] 0.44, 95% confidence interval [CI]: 0.23–0.83) or with URRMI (OR 0.61, 95% CI: 0.39–0.92) were less likely to be in the malignant tumor class than in the other sporadic disease class. Older adults aged between 75 and 84 years (OR 1.89, 95% CI: 1.35–2.63) were more likely to be in the malignant tumor class than in the other sporadic disease class.

#### Cerebrovascular diseases versus other sporadic diseases

Older adults aged between 75 and 84 years (OR 0.70, 95% CI: 0.53–0.94) or with URRMI (OR 0.62, 95% CI: 0.42–0.92) were less likely to be in the cerebrovascular diseases class than in the other sporadic disease class. Older adults aged 85 years or over (OR 1.48, 95% CI: 1.05–2.07) were more likely to be in the cerebrovascular diseases class than in the other sporadic disease class.

#### Ischemic heart disease versus other sporadic diseases

Female patients (OR 0.72, 95% CI: 0.54–0.97) or patients with URRMI (OR 0.52, 95% CI: 0.31–0.86) were less likely to be in the ischemic heart disease class than in the other sporadic disease class. Older adults aged 85 years or over (OR 1.53, 95% CI: 1.02–2.31) were more likely to be in the ischemic heart disease class than in the other sporadic disease class.

#### Arthrosis versus other sporadic diseases

Female patients (OR 2.22, 95% CI: 1.67–2.95) were more likely to be in the arthrosis class than in the other sporadic disease class.

### Total aggregate and average per patient spending, for latent classes

**Fig. 1:**
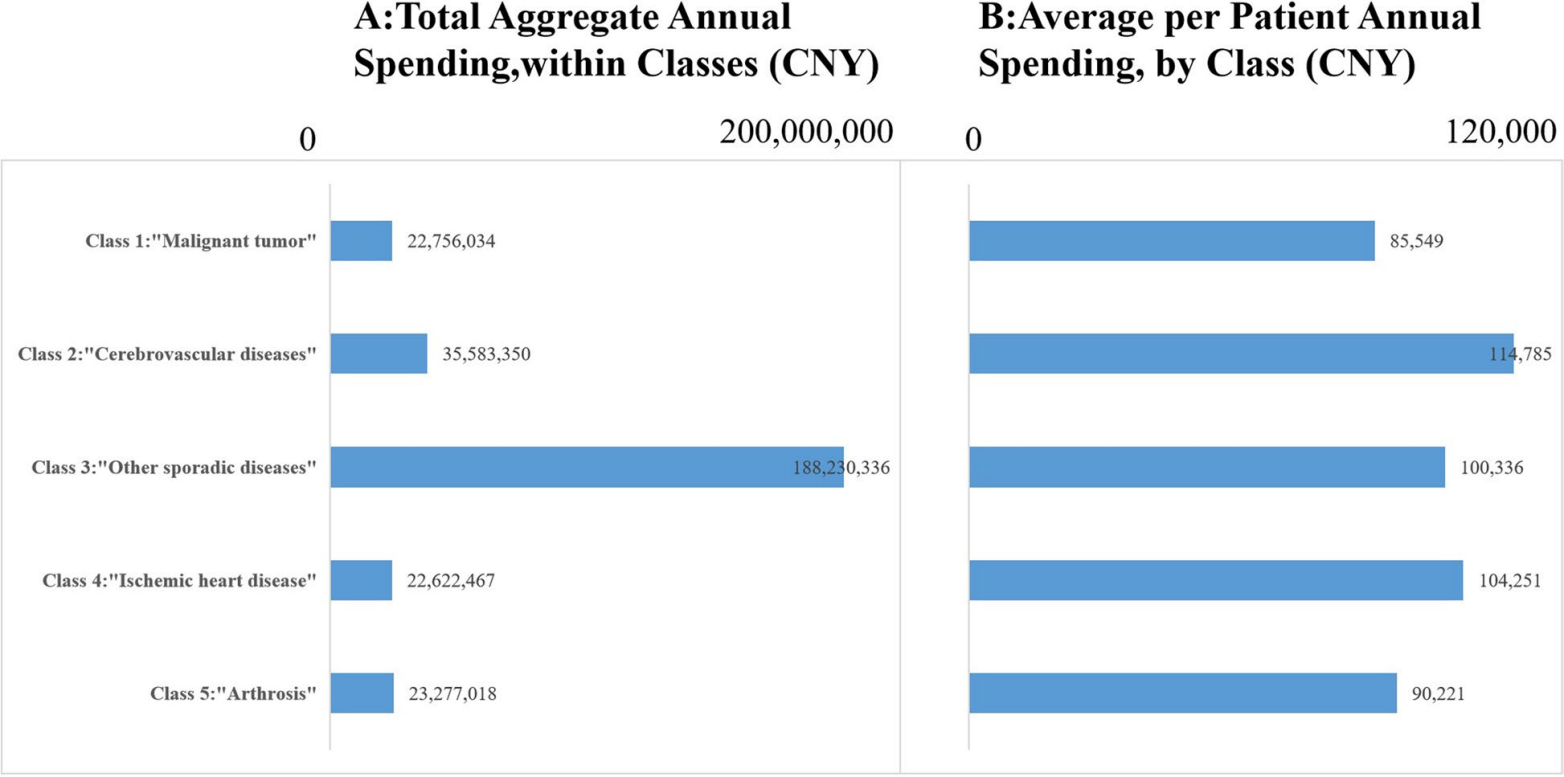
Total aggregate (Panel **A**) and average per patient (Panel **B**) spending, for latent classes, 2019

Figure 1 presents the results of total aggregate and average per patient spending, for latent classes. Total aggregate spending is greatest in class 3, due to the large class size. Average per patient annual spending is greatest in class 2 “cerebrovascular disease”, and is least in class 1.

## Discussion

Using the public medical insurance claimants’ data in Shanghai, China, we took a cross-sectional analysis of the top 1% older adults and identified five meaningful patterns of clinical conditions. We also explored the associations between characteristics of high-cost older adults and patterns of clinical conditions. The finding provides policy implications on disease burden cost, disease prevention, and care management not only for China, but also for other developing countries facing the challenge of rapid population aging.

In this study, we applied LCA to high-cost older adults and identified five empirically derived subgroups of clinical conditions. Four single-condition subgroupings of high-cost older adults accounted for relatively small proportions. The “other sporadic diseases” subgroup comprised most (64.1%) high-cost older adults. Among four single-condition subgroupings, we found similar portions, with the “cerebrovascular diseases” subgroup having the highest proportion (10.6%), followed by the “malignant tumor” (9.1%), “arthrosis” (8.8%), and “ischemic heart disease” (7.4%) subgroups. To the best of our knowledge, this was the first study using social medical insurance claims data to define meaningful subgroups of high-cost older adults in China. Identification of these clinical condition-based subgroups may help policy makers to understand the diverse characteristics of high-cost older adults and deliver tailored interventions for each subgroup.

The subgroups shared a number of similarities with other typologies, particularly that described by Buja et al. [8] who focused on a cohort of high-need, high-cost elderly patients and identified five disease clusters: “metabolic-ischemic heart diseases,” “neurological and mental disorders,” “cardiac diseases such as congestive heart failure and atrial fibrillation,” “respiratory conditions,” and “neoplasms.” Similar to the present study, Davis et al. [6] identified seven classes among high-cost adults, namely, “neurologic and catastrophic conditions,” “diabetes with multiple comorbidities,” “acute illness superimposed on chronic conditions,” “end-stage renal disease,” “conditions requiring highly specialized care,” “cardiopulmonary conditions,” and “patients with few comorbidities.” Detailed comparisons with subgroups of high-cost patients are limited due to different data sources and different definitions of high-cost health care users [5].

A notable finding from this analysis is that the largest proportion of high-cost older adults were classified into the subgroup defined as “other sporadic diseases,” indicating complex and multiple comorbid conditions. This finding has important economic and policy implications for health care systems. Older adults with multiple clinical conditions have complex health care needs [8] and are the target population to improve savings in costs of care. An integrated care approach involving complex case management, disease management, and care coordination is needed to develop interventions to manage high and costly health care use [5]. Our findings support policy makers focusing on these complex older patients in developing clinical practice guidelines [24] to improve evidence-based management.

Single-condition subgroups also have policy implications. In this data-driven analysis, “cerebrovascular diseases,” “malignant tumor,” “arthrosis,” and “ischemic heart disease” emerged as distinct subgroups. These conditions matched with the increasing rate of disease incidence. Cardiovascular disease and cancer were the top two causes of death in China [25]. From 2015 to 2020, the total incidence of cancer in China increased from 3.9 million to 4.6 million [26]. The top two diseases of prevalence among middle-aged and elderly people in China were hypertension, arthritis or rheumatism [27]. Such disease profiles may be partly due to the rapid socioeconomic development and urbanization in China in the past decades [26, 28]. It is worth to mention that the claim data may under-estimate the situation, as some care which is not covered by the public medical insurance is not recorded. Our finding suggests that better healthcare policies are required to enhance disease prevention, early detection, and effective treatments to reduce the healthcare expenditure in the long run. These subgroups should be the focus of future work to improve health system efficiency in managing high-cost older adults. Although previous intervention studies aiming to limit unnecessary high health care utilization among patients with cerebrovascular diseases have shown considerable heterogeneity, some robust evidence is important, such as team-based care models, support for transitional care, and use of post-acute rehabilitation [29]. For cancer patients, previous research indicates that cost drivers (such as prescription medications, office-based visits, and inpatient hospitalization) may differ by types of cancer [30], and admission to the intensive care unit is associated with high costs [31]. The economic burden of musculoskeletal diseases is also reported as a public concern with population aging [32]. In our study, we focused on the top 1% of social medical insurance beneficiaries, and ischemic heart disease was identified as a cluster among high-cost older adults, which was consistent with a previous study focusing on older adults with complex health care needs [8]. Our study represents an initial step to understand high-cost older adults by each segment to inform the development of tailored interventions and policy measures.

Our results support some identifiable predictors of these subgroups. Age, sex, and type of medical insurance were predictors of subgroups in high-cost older adults. Compared with the “other sporadic diseases” subgroup, older adults aged between 75 and 84 years were more likely to be the “malignant tumor” subgroup, and those aged 85 years and over were more likely to be the “cerebrovascular diseases” and “ischemic heart disease” subgroups. Our finding is consistent with the fact that a disproportional burden of cancer occurs in people aged 65 years and over [33]. In addition, our results share a number of similarities with Jin et al.’s findings that the prevalence of cardiovascular disease increased with age [34]. Women were more likely to be in the “arthrosis” subgroup. This was in good agreement with Oh and Yoon’s finding that the economic burden of musculoskeletal disease per person was heaviest for aged females [32]. Older adults with UEMI were more likely to be in the “cerebrovascular diseases,” “malignant tumor,” and “ischemic heart disease” subgroups, as compared with the “other sporadic diseases” subgroup. These findings may provide implications for age- and sex-specific interventions in each subgroup. Another policy consideration supported by our findings is to narrow the potential benefit inequality between UEMI and URRMI to improve the integration of urban–rural medical insurance systems in China [35]. Given that we only include three covariates (sex, age, insurance type) in our regression analysis due to data limitation, the results from such analysis should thus be treated with considerable caution. Future studies are warranted to consider other confounders, such as socioeconomic status, health status, health behavior, and residence.

Our findings provide initial directions for the development of appropriate care management and intervention strategies for high-cost older adults. Future research and policy actions should focus on the identified subgroups to develop tailored clinical guidelines [36], person-centered integrated care models [37, 38], and high-value health care [39]. For example, published studies have shown that arthroscopy for osteoarthritis and percutaneous coronary intervention for stable coronary disease are examples of where quality improvement initiatives must focus on encouraging providers to do less [40].

Our research suggests that it is important for healthcare policy makers to pay attention to these subgroups of high-cost older adults, to develop tailored interventions to improve health care services efficiently and effectively. Policy implications may include improving early screening and detection of cardiovascular disease and cancer related risk factors, enhancing care management for older adults with complex needs, and exploring alternative payment approaches to combat rising medical spending.

### Limitations

Several limitations should be noted. First, we focused on the top 1% of patients. It is common to define high-cost health care users, as has been widely used in prior studies [5, 6]. Still, in future studies, we encourage the use of multiple definitions to capture the complexity of high-cost older adults. Second, the use of claims data may mean that some conditions are under-represented [41]. For example, there may be insufficient data on neurological and mental disorders, which have been reported to represent a large economic burden in China [42, 43]. We did not include this condition as they were rare in database. China’s health care budget allocation was heavily skewed towards somatic diseases, while less than 1% of the total health expenditure was spent on mental disorders [44]. Third, we only used primary diagnosis to predict high-cost users, which could not reflect the co-morbidity conditions which were common in older adults [8]. While it is common to use mutually exclusive clinical conditions in prior literature [45]. Still, we encourage future studies to include and yield further data, for example, the combination of primary diagnosis with procedure diagnosis [46] to better describe these complex profiles. Finally, we only analyzed data from the Shanghai Basic Social Medical Insurance Database, which limited the generalizability of the results to other areas of China.

### Strengths

To the best of our knowledge, this was the first study using social medical insurance claims data to define meaningful subgroups of high-cost older adults in China. We used a novel person-centered method, LCA, to identify patterns of clinical conditions within a representative sample of Shanghai Social Medical Insurance beneficiaries, which can be replicated in other systems to support the management of subgroups of high-cost older adults.

## Conclusions

This study advances the understanding of profiles among high-cost older adults, distinguishing five meaningful patterns based on clinical conditions in high-cost older adults. Age, sex, and type of medical insurance were predictors of high-cost older adult subgroups. Profiling patterns of clinical conditions among high-cost older adults is potentially useful as a first step to inform the development of tailored management and interventions strategies. Additional efforts are needed to focus on subgroups and design policy to optimize health care systems.

## Supplementary Information

**Additional file 1: Supplementary Figure 1.** Selection of high-cost older adults.

**Additional file 2: Supplementary Table 1.** Selected clinical conditions and related ICD-10 codes.

## Data Availability

Aggregate data available upon request.

## Abbreviations

LCA: Latent class analysis
UEMI: Urban Employees’ Medical Insurance
URRMI: Urban and Rural Residents’ Medical Insurance
