# Supplementary figures and images for: Identifying patterns of clinical conditions among high-cost older adult health care users using claims data: a latent class approach
Source: Int J Equity Health. 2022 Jun 20;21:86. doi: 10.1186/s12939-022-01688-3 (PMC9210624; doi:10.1186/s12939-022-01688-3)

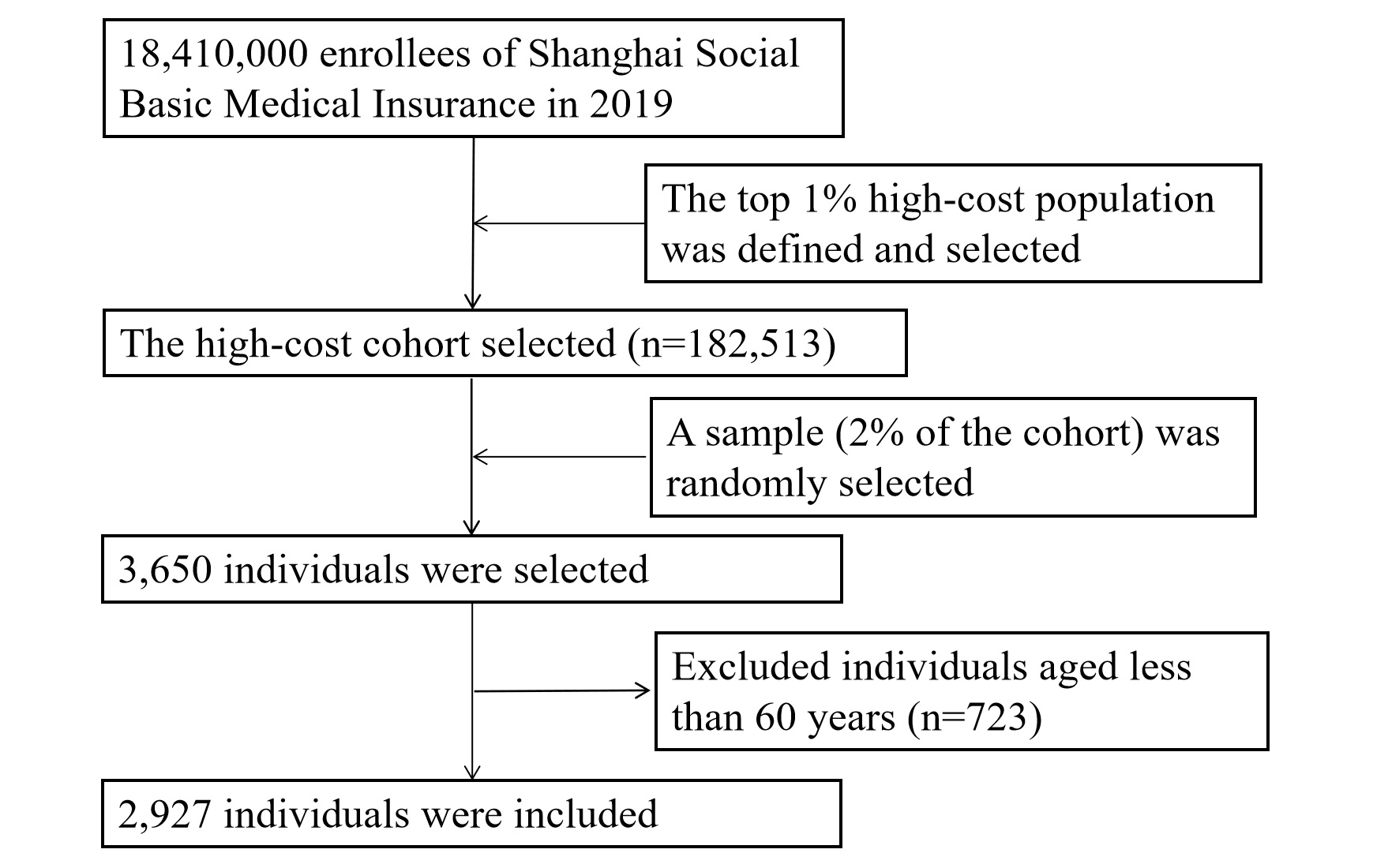

Supplement: Supplementary file 1 — Additional file 1: Supplementary Figure 1. Selection of high-cost older adults. [file 12939_2022_1688_MOESM1_ESM.jpg]
