# Supplementary material for: Identifying patterns of clinical conditions among high-cost older adult health care users using claims data: a latent class approach
Source: Int J Equity Health. 2022 Jun 20;21:86. doi: 10.1186/s12939-022-01688-3 (PMC9210624; doi:10.1186/s12939-022-01688-3)
Supplement: Supplementary file 2 — Additional file 2: Supplementary Table 1. Selected clinical conditions and related ICD-10 codes. [file 12939_2022_1688_MOESM2_ESM.docx]

Supplementary Table 1. Selected clinical conditions and related ICD-10 codes

| System | Clinical Conditions | Related Codes |
| --- | --- | --- |
| Endocrine System | Thyroid Disease | E00 – E07 |
|  | Diabetes | E10 – E14 |
| Circulatory System | Hypertension | I10 – I15 |
|  | Ischemic heart disease | I20 – I25 |
|  | Other types of heart disease | I26 – I52 |
|  | Cerebrovascular diseases | I60 – I69 |
|  | Other vascular diseases | I70 – I87 |
| Respiratory System | Lung and bronchial diseases | J00 – J98 |
| Skeletal System | Arthrosis | M00 – M25 |
|  | Spondylosis | M45 – M49 |
|  | Other back diseases (related to intervertebral discs) | M50 – M54 |
| Urinary System | Nephritis | N02 – N05; N10 – N13 |
|  | Renal failure | N17 – N19 |
|  | Stones | N20 – N23 |
| Digestive System | Gastric diseases | K21 – K31 |
|  | Intestinal diseases | K55 – K63 |
|  | Liver diseases | K70 – K77 |
|  | Biliary and pancreatic diseases | K80 – K87 |
| Cancer | Malignant tumor | C00 – C97 |
|  | Benign tumor | D00 – D36 |
